# Body size has primacy over stoichiometric variables in nutrient excretion by a tropical stream fish community

**DOI:** 10.1038/s41598-022-19149-w

**Published:** 2022-09-01

**Authors:** Priscila Oliveira-Cunha, Peter B. McIntyre, Vinicius Neres-Lima, Adriano Caliman, Beatriz Moreira-Ferreira, Eugenia Zandonà

**Affiliations:** 1 Programa de Pós-Graduação em Ecologia e Evolução, Universidade do Estado do Rio de Janeiro, Rua São Francisco Xavier, 524, Maracanã, Rio de Janeiro, RJ CEP 20550-013 Brazil; 2 Department of Natural Resources and the Environment, Cornell University, Ithaca, NY USA; 3 Departamento de Ecologia, Universidade Federal do Rio Grande Do Norte, Natal, RN Brazil; 4 Departamento de Ecologia, Universidade do Estado do Rio de Janeiro, Rio de Janeiro, RJ Brazil

**Keywords:** Ecology, Biogeochemistry, Ecosystem ecology, Freshwater ecology, Tropical ecology

## Abstract

Ecological Stoichiometry (ES) and the Metabolic Theory of Ecology (MTE) are the main theories used to explain consumers’ nutrient recycling. ES posits that imbalances between an animal’s body and its diet stoichiometry determine its nutrient excretion rates, whereas the MTE predicts that excretion reflects metabolic activity arising from body size and temperature. We measured nitrogen, phosphorus and N:P excretion, body N:P stoichiometry, body size, and temperature for 12 fish species from a Brazilian stream. We fitted competing models reflecting different combinations of ES (body N:P, armor classification, diet group) and MTE (body size, temperature) variables. Only body size predicted P excretion rates, while N excretion was predicted by body size and time of day. N:P excretion was not explained by any variable. There was no interspecific difference in size-scaling coefficients neither for N nor for P. Fitted size scaling coefficients were lower than the MTE prediction of 0.75 for N (0.58), and for P (0.56). We conclude that differences in nutrient excretion among species within a shared environment primarily reflect contrasts in metabolic rates arising from body size, rather than disparities between consumer and resource stoichiometry. Our findings support the MTE as the primary framework for predicting nutrient excretion rates.

## Introduction

Nutrients are chemical elements essential to life. They are fundamental constituents of all organisms and integrate a series of biomolecules indispensable for growth, homeostasis and reproduction of organisms^1^. Nutrients transit between abiotic (i.e. soil, water, atmosphere) and biotic compartments of ecosystems through anabolic and catabolic processes, such as primary production and excretion, respectively. Nutrients such as nitrogen (N) and phosphorus (P) are generally found in limiting amounts for primary producers in many ecosystems^2^ and, therefore, understanding the mechanisms and pathways that mediate the recycling and the absolute and relative availability of N and P to organisms is critical to understanding the functioning of ecosystems. In this context, consumer nutrient recycling of N and P can have a significant impact in their ecosystems^3^. Through their ingestion, elimination and transport of nutrients between habitats, consumers can have a direct influence on the nutrient cycling process and can act as sinks or sources of nutrients depending on the context^3–5^.

Two distinct conceptual frameworks have been proposed to understand and predict differences among consumers in nutrient recycling rates: Ecological Stoichiometry (ES) and the Metabolic Theory of Ecology (MTE). ES is based on the premise that consumer body composition is homeostatic, even when the nutrient content of the diet varies widely^1,4^. Therefore, ES predicts that the rates and ratios of excreted nutrients reflect imbalances between the makeup of the diet versus body tissues^1^. For example, individuals with a nutrient-rich diet should excrete more nutrients than counterparts with a nutrient-poor diet. By extension, individuals with high dietary N:P should excrete more N than those with a low N:P diet. Differences in body composition should create similar disparities in excretion, such as the high P demand for growing bones^4,6,7^ reducing the P excretion rates of vertebrates.

The MTE posits that metabolic rates are the primary determinant of all ecological processes, from individual to ecosystem scales^8,9^. Metabolic rates are affected by body size and ambient temperature^8^. The relation between metabolism and body size is a power function with a scaling coefficient of ¾^8,10^, which means that smaller animals excrete disproportionately more nutrients per unit body mass than larger animals^4,11,12^. Allgeier et al.^13^ found evidence of ¾-power scaling for N and P excretion of fish and macroinvertebrates in marine ecosystems, whereas Vanni and McIntyre^14^ found lower scaling coefficients for both nutrients across all types of aquatic animals. Temperature also holds a central place in the MTE because it mediates the rates of chemical reactions^8,15^; metabolic rates increase exponentially with temperature, hence nutrient excretion rates should also be positively related to temperature.

Both ES and MTE are compelling frameworks because they are rooted in fundamental principles, yet they emphasize completely different predictors of nutrient recycling due to their contrasting emphases on elemental mass balance versus energetics^13,14,16^. Species-rich ecosystems provide an interesting arena in which to compare ES- and MTE-based predictors of nutrient excretion rates because species vary widely in diet, tissue composition, and body size^13,17^. Direct comparisons of the explanatory power of ES and MTE for aquatic animal excretion rates have included large numbers of coastal marine species^13^ as well as a literature synthesis across a host of aquatic vertebrates and invertebrates^14^. Both of those studies concluded that MTE has primacy over ES because body size was more important than diet or consumer nutrient content. However, the 5–8 orders of magnitude range in body sizes tested in each study may have obscured the comparatively modest range of stoichiometric variation^18^. Thus, it is possible that the influence of ES on nutrient excretion by consumers might become more apparent when focusing on one taxonomic group in a single ecosystem. Therefore, in this study, we focused on distantly related fish species from one rainforest stream as opposed to several taxonomic orders of reef fish and macroinvertebrates^13^ and aquatic vertebrates and invertebrates from multiple ecosystems^14^.

We compared the predictive power of the ES and MTE frameworks using nutrient excretion rates from 12 species in the fish community of a Neotropical stream. Fishes play a significant role in nutrient cycling by virtue of storing large quantities of phosphorus in their tissues^4,5^, transporting nutrients between habitats^4,19^, varying widely in dietary and body nutrient content^20,21^, and being abundant in many freshwater ecosystems^22,23^. Our study species ranged in body size from 0.02 to 22.0 g dry mass, and we used substantial seasonal variation in water temperature to test its effects on excretion rates. These fish species also differed sharply in the nutrient content of their diet (from algivory/detritivory to piscivory) and their body tissues (3 of 12 species are armored catfish, which are renowned for high body P). By collecting all data from a single site with consistent methods and background environmental conditions (flow, nutrient levels), our survey of excretion rates was designed to offer a rigorous comparison of the influence of ES and MTE variables.

We expected to find support for both the ES and MTE frameworks, and we adopted a testing-based procedure model-selection approach to jointly test their influence. We made the following four predictions: (1) Fish from higher trophic positions should excrete more N and P than like-sized fish from lower trophic positions due to the general increase in dietary nutrient content with trophic level^23,24^. (2) Armored catfish should excrete less P than like-sized fishes due to their high P demand for building their boney armor. (3) Nutrient excretion rates should increase with water temperature, reflecting higher resting metabolic rates. (4) The relationship between body size and nutrient excretion should be allometric with a scaling coefficient of ~ 0.75, in accordance with the MTE.

## Results

Nitrogen excretion rates ranged from 1.8 to 2667.3 µg NH_4_-N ind^−1^ h^−1^, while phosphorus excretion rates ranged from 0.015 to 117.8 µg P ind^−1^ h^−1^. The smallest species (*P. harpagos* and *M. microlepsis*) had the lowest average N and P excretion rates per capita, while larger species (*R. quelen* and *Rineloricaria* sp.) had the highest per capita N and P excretion rates. Excretion rates scaled allometrically with body mass, as indicated by scaling coefficients smaller than 1. Indeed, the average mass-specific excretion rates of the smallest species were, in general, the highest (Table 1).

**Table 1:** Mean per capita excretion rates (µg ind^−1^ h^−1^) and mass-specific excretion rates (µg g^−1^ h^−1^) of NH_4_-N and SRP-P of all studied fish species.

Species	Dry weight (g)	N excretion rate		P excretion rate		Mass-specific N excretion rate	Mass-specific P excretion rate
	Mean ± SD	n	Mean ± SD	n	Mean ± SD	Mean ± SD	Mean ± SD
*P.harpagos*	0.10 ± 0.61	24	40 ± 22	12	2.3 ± 2.4	466.0 ± 271.0	36.7 ± 38.0
*M.microlepis*	0.18 ± 0.14	15	54 ± 31	10	3.9 ± 4.5	385.0 ± 163.0	22.2 ± 22.7
*A. leptos*	0.41 ± 0.03	6	98 ± 40	5	8.6 ± 6.7	174.0 ± 90.7	6.4 ± 3.6
*C.vidali*	0.58 ± 0.24	21	109 ± 67	13	6.6 ± 5.8	179.0 ± 78.0	12.2 ± 10.8
*Trichomycterus sp*	0.89 ± 0.76	5	78 ± 56	4	11.1 ± 8.9	139.0 ± 65.5	14.5 ± 15.6
S.barbatus*	1.19 ± 0.42	23	138 ± 94	15	13.9 ± 15.2	95.1 ± 56.2	6.1 ± 5.2
*P.lateristriga*	1.43 ± 1.20	22	115 ± 97	12	12.4 ± 8.8	107.0 ± 97.7	14.1 ± 8.9
Rineloricaria sp*	2.51 ± 2.08	20	197 ± 149	14	25.6 ± 26.5	91.2 ± 38.7	6.7 ± 4.8
*B. ornaticeps*	2.94 ± 1.95	19	197 ± 169	8	9.7 ± 13.2	149.0 ± 176.0	30.5 ± 47.0
A.multispinis*	2.98 ± 2.35	8	130 ± 113	3	4.1 ± 3.8	59.5 ± 30.5	3.3 ± 1.6
*S.marmoratus*	4.61 ± 0.42	12	135 ± 124	6	9.5 ± 3.3	33.6 ± 22.3	9.1 ± 11.2
*R.quelen*	6.88 ± 8.41	18	494 ± 753	9	24.9 ± 40.0	103.0 ± 74.3	2.5 ± 2.2

*Armored catfish—fish of the family Loricariidae and Callichthyidae, named for the rows of overlapping bony plates that cover and protect their bodies.

**Figure 1:**
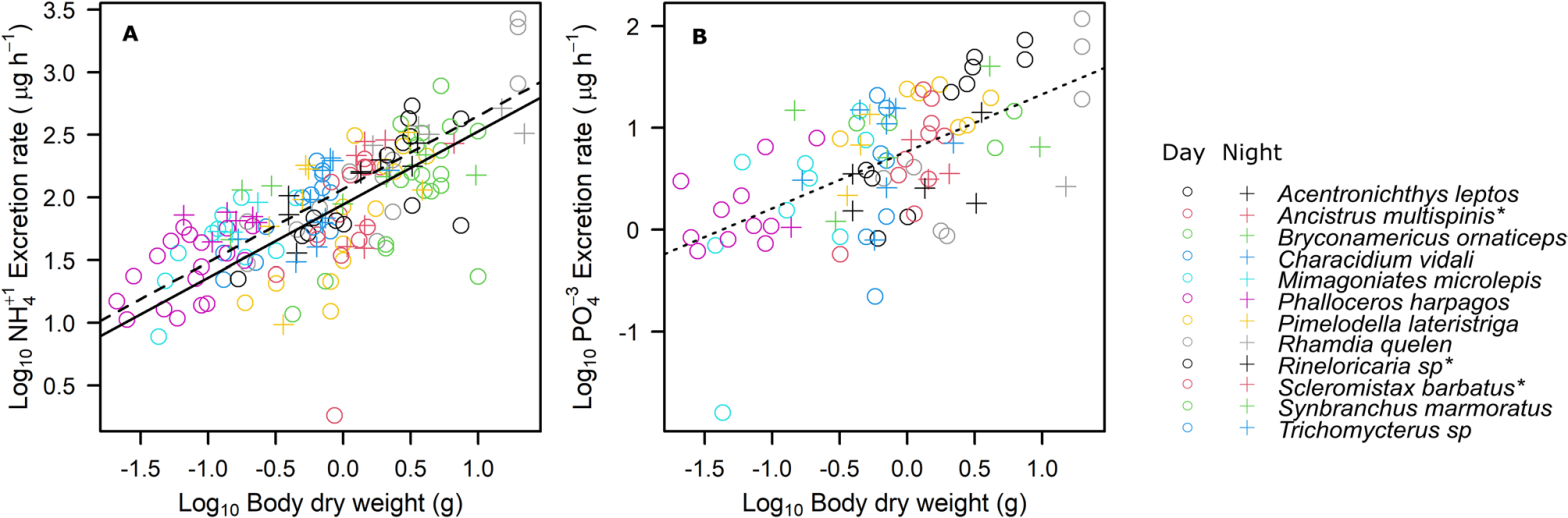
Fitted allometric relationships between fish body mass and excretion rates for N (**A**) and P (**B**) across 12 species in a Brazilian stream. In (**A**), the solid line represents the best fit for daytime excretion rates, and the dashed line represents nighttime excretion rates. There was no effect of time of day in the P excretion rate. Different colors represent different species as indicated in the legend.

**Figure 2:**
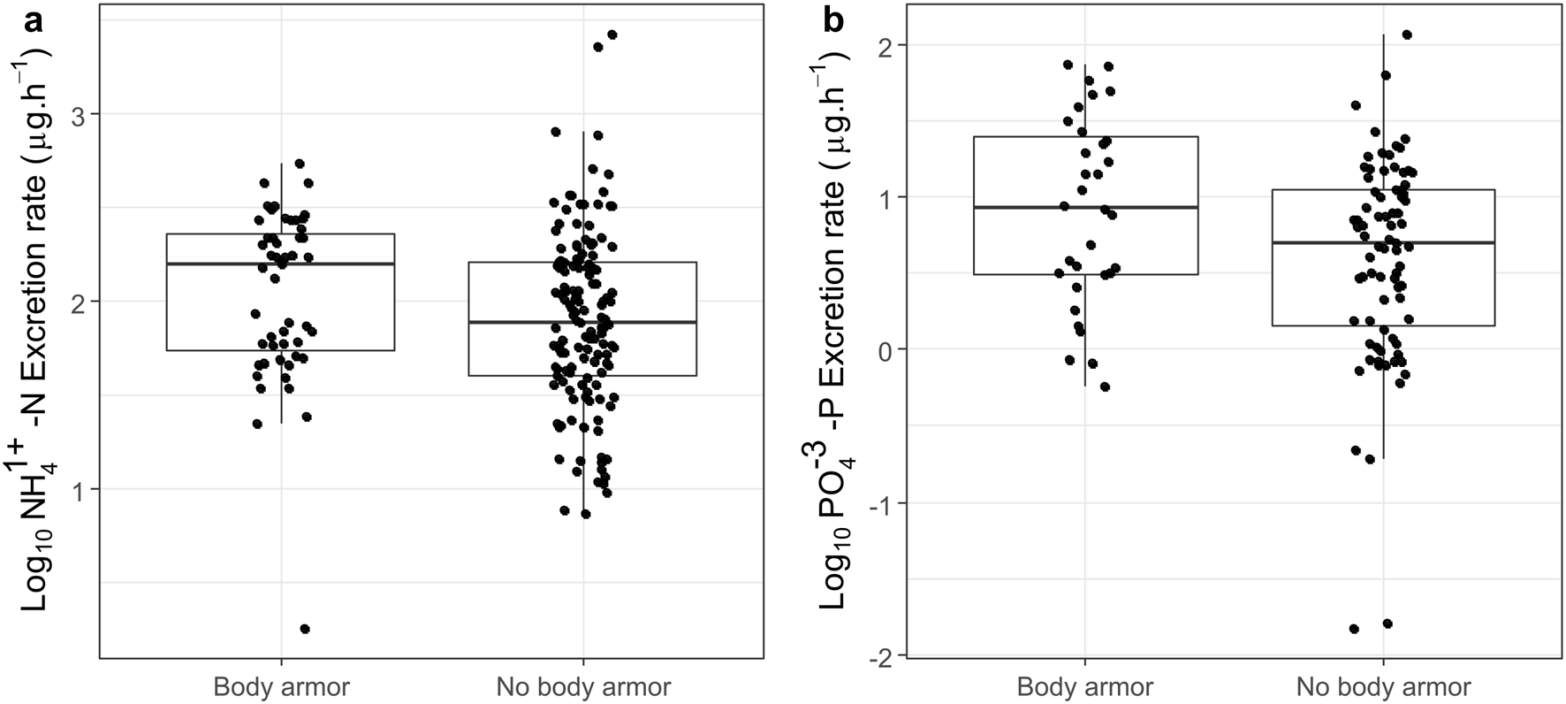
Excretion rates of (**a**) N and (**b**) P in armored versus non-armored fish species.

The best model selected to explain N excretion rates was the one that contained the predictors body size and time of day (Table 2, Fig. 1). The N excretion rate at night was slightly higher than during the day. The mean difference between the overnight excretion rate and the daytime excretion rate was 1.33 (CI_95%_ = 1.05–1.68) µg NH_4_-N ind^−1^ h^−1^. The best model for P excretion was the one including only body size as a predictor (Table 2, Fig. 1). Armored and non-armored species differed significantly in their body nutrient composition, particularly for P (Fig. S1 in the Supplementary Material). But even if armored fish had higher body P content (Fig. S1), armor classification had no significant effect on excretion rates (p > 0.05) (Fig. 2). None of the predictors had a significant effect on excreted N:P ratios. Thus, any of the stoichiometric variables nor temperature explained N, P excretion rates or N:P excretion ratio.

**Table 2:** Pairwise comparison of models fitted to N and P excretion rates for fish in a Brazilian stream.

Model	More complex model	Simplest model	χ^2^	p-value
**Log**_**10**_** NH**_**4**_** with body N:P**				
	Body size + Temperature + Body NP + FG + TD	Body size + Temperature + FG + TD	0.585	0.444
	Body size + Temperature + FG + TD	Body size + Temperature + TD	2.543	0.468
	Body size + Temperature + TD	Body size + TD*	1.060	0.303
**Log**_**10**_** NH**_**4**_** with Armor classification**				
	Body size + Temperature + Armor + FG + TD	Body size + Armor + FG + TD	1.235	0.267
	Body size + Armor + FG + TD	Body size + Armor + TD	4.471	0.215
	Body size + Armor + TD	Body size + TD*	0.806	0.369
**Log**_**10**_** PO**_**4**_** with body N:P**				
	Body size + Temperature + Body NP + FG + TD	LDW + Temperature + FG + TD	3.124	0.077
	Body size + Temperature + FG + TD	LDW + Temperature + TD	5.485	0.140
	Body size + Temperature + TD	LDW + TD	2.576	0.109
	LDW + TD	Body size*	0.431	0.512
**Log**_**10**_** PO**_**4**_** with Armor classification**				
	Body size + Temperature + Armor + FG + TD	Body size + Armor + FG + TD	3.450	0.063
	Body size + Armor + FG + TD	Body size + Armor + FG	1.638	0.201
	Body size + Armor + FG	Body size + Armor	7.454	0.059
	Body size + Armor	Body size*	0.290	0.590

We used the testing-based procedures based on backward elimination and Chi-square test to select the final model. The variables Body NP, Armor and Feeding Group (FG) are the predictors for the Ecological Stoichiometry Theory, while Body size and Temperature are the predictors for the Metabolic Theory of Ecology. Time of day (TD) refers to the period of day (daytime or nighttime) in which the fish incubation took place. * Indicates the final selected models.

The overall scaling coefficient between N excretion rates with body mass was 0.58 (CI_95%_ = 0.47–0.70), which was significantly less than 0.75 (Table 3). The variance explained by body mass (marginal R^2^) was 58%, while the variance explained by the entire model (conditional R^2^), including both fixed (body mass) and random (species/family) effects was 65%, indicating that differences between species and family explained some of the variation in N excretion rates. We found no difference between the model with random intercept and the model with random intercept and slope (χ^2^ = 1.19, p-value = 0.88). This indicates that the scaling coefficient of the relationship between N excretion rate and body size does not differ significantly between species (Table 4). For P excretion, the overall scaling coefficient was 0.56 (CI_95%_ = 0.41–0.74), which was slightly different from the 0.75 of the MTE prediction (Table 3). The marginal R^2^ and conditional R^2^ were identical (36%), indicating that accounting for species identity did not increase the explanatory power of the model. The species-specific scaling coefficients for P excretion were all comparable and there was no difference between the model with random intercept and the model with random intercept and slope (χ^2^ = 0.005, p-value = 1.00) (Table 4).

**Table 3:** Fitted parameters of the final selected model for N and P excretion rates.

Excretion rate	Fixed effects	Estimate	SE	df	T-value	p-value
**Log**_**10**_** NH**_**4**_						
	Intercept	1.943	0.053	4.2	36.363	< 0.001
	Log_10_ Body size	0.584	0.049	65.2	11.807	< 0.001
	Time of Day _Night_	0.123	0.052	158.2	2.392	0.018
**Log**_**10**_** PO**_**4**_						
	Intercept	0.768	0.054	91.0	14.247	< 0.001
	Log_10_ Body size	0.561	0.078	91.0	7.177	< 0.001

**Table 4:** Intercept and coefficient values (slope) from the relationship between body size (g) and N and P excretion rates (µg ind^−1^ h^−1^) for all fish species.

Species	N excretion			P excretion	
	Intercept	Log_10_Body size	Time of the day: Night	Intercept	Log_10_Body size
*Acentronichthys leptos*	1.94	0.58	0.12	0.77	0.56
Ancistrus multispinis*	1.90	0.58	0.12	0.77	0.56
*Bryconamericus ornaticeps*	1.96	0.58	0.12	0.77	0.56
*Characidium vidali*	1.98	0.58	0.12	0.77	0.56
*Mimagoniates microlepis*	2.02	0.58	0.12	0.77	0.56
*Phalloceros harpagos*	2.02	0.58	0.12	0.77	0.56
*Pimelodella lateristriga*	1.87	0.58	0.12	0.77	0.56
*Rhamdia quelen*	2.01	0.58	0.12	0.76	0.56
Rineloricaria sp*	1.96	0.58	0.12	0.77	0.56
Scleromystax barbatus*	1.89	0.58	0.12	0.77	0.56
*Synbranchus marmoratus*	1.81	0.58	0.12	0.77	0.56
*Trichomycterus sp*	1.95	0.58	0.12	0.77	0.56

*Armored catfish—fish of the family Loricariidae and Callichthyidae, named for the rows of overlapping bony plates that cover and protect their bodies.

## Discussion

Metabolic Theory of Ecology (MTE) and Ecological Stoichiometry (ES) are two common frameworks used to predict energy and nutrient budgets at various biological levels of organization^1,8^. By comparing both theories, we found that the ES variables (diet, body N:P, “armor”) were outperformed by body size, which indicates that the ES framework has relatively little predictable effect on nutrient excretion compared to the role of body size. Also, we saw that the scaling coefficients for the relation between N excretion and body size were lower than the 0.75 coefficient predicted by MTE.

Similarly to previous studies, our results show that body size is a key control on excretion of N and P by fish^13,25–27^. Bigger fish excreted more nutrients per capita when compared to smaller fish, however they excreted less nutrients per mass than smaller fish. This result was expected since MTE states that there is an allometric relation between metabolism and body size, described by ¾-power scaling^8^. In fact, Allgeier et al.^13^ found quantitative support for ¾-power scaling of nutrient excretion rates with body mass using data from marine fish and invertebrates. In our study, the lower scaling coefficients for both N and P were closer to a ^2^/_3_ factor than ¾, perhaps echoing debates in metabolic ecology about the most appropriate scaling factor^28–30^. Our results are similar to Vanni and McIntyre^14^, who found scaling coefficients comparable to ours (0.68 for N; 0.56 for P). The ecological significance of these low scaling coefficients is that size-based increases in nutrient excretion are smaller than would be expected.

The reasons for these lower-than-expected scaling coefficients are uncertain because the ingestion and assimilation of nutrients should be directly related to metabolic rates, hence it is reasonable to expect that release of nutrients in wastes would be as well. One possibility is that focusing on dissolved wastes while excluding solid wastes could create a bias in studies like ours^14^. Alternatively, biochemical mechanisms have received little attention. For instance, Delong et al.^31^ found a gradient of size-scaling coefficients from 1.0 to 0.75 in a survey of metabolism across prokaryotes, unicellular eukaryotes and metazoans, and argued that the differences reflected the number of membrane-bound sites where ATP synthesis and proton pumping occur, as well as differential constraints on resource supply and vascular systems. Although MTE refers to metabolism instead of excretion, it is reasonable to assume the theory also applies to any biological rate that is derived from metabolism^14^. Therefore, it could be that our lower than ¾ power-scaling is related to other fish characteristics, such as fish growth and ingestion rates or to ontogenetic diet shifts and sex.

A novel aspect of our study was the range of temperature variation during our excretion incubations (9.9 to 25.7 °C), and we were surprised to find no evidence that temperature affects nutrient excretion rates of fish. Both the MTE framework and many previous studies^14,32–36^ have suggested that temperature should have discernible effects. This is a surprising result since fish are poikilotherms, which means their body temperature is determined by the external temperature of the water they inhabit and should have direct influence on metabolic rates, feeding rates and activity levels^37^. It could be that in the tropics, as the rate of diel temperature change is usually slow^13,38^, fish can acclimate and perform metabolic compensation^37^. Consequently, because of fish acclimation, we see no apparent changes in nutrient excretion rates.

As for the effects of ES variables, diet and body stoichiometry, our results revealed counterintuitive patterns. Many studies have demonstrated that diet can directly influence fish nutrient excretion rates^39,40^. The nutritional quality of the diet of aquatic consumers progressively increases from detritivores, to omnivores, to invertivores and, finally, to piscivores^24^. Therefore, according to ES, it is expected that piscivores present the highest nutrient excretion rates compared to detritivores, for example. However, similarly to other comparisons of MTE and ES variables^13,14^, our results do not reflect this pattern. Vanni and McIntyre^14^ attribute the lack of a diet effect to the absence of information on growth, ingestion, and egestion data, and Allgeier et al.^13^ question how useful diet is for predicting nutrient excretion rates. Clearly, we need future studies to investigate the importance of diet by measuring growth, ingestion, excretion and egestion rates across a range of feeding and taxonomic groups.

As expected, armored catfish species presented up to 3 × more P in their body composition than the other fish species. Therefore, according to ES predictions, it was expected that they would excrete less P because of their higher P demand for building their boney plates. However, armored and non-armored fish did not differ in their P excretion rates. One possible explanation for this deviation from our prediction is that we sampled primarily adult fishes whose bony skeletons have already been formed, such that further assimilation of dietary P reflects only tissue maintenance. Perhaps if we had sampled individuals in different life stages, we would see growing individuals with a higher P demand and consequent low P excretion.

The armor classification captures the major differences in body composition among our study species. Using the armor classification did not significantly differ from using body NP (Table 2), so relying on a simple classification of armor investment by fish could be a sufficient proxy for differences in body stoichiometry. Given that directly measuring body composition is both time-consuming and requires specialized lab facilities, the use of such proxies is appealing in lieu of systematic characterization of body P and stoichiometry across aquatic animals.

We found that fish P excretion rates did not differ between day or night, however fish N excretion rates were higher during the night. These results are similar to what was found by Oliveira-Cunha et al.^38^, where the N excretion of two fish and one shrimp species were influenced by the time of day, which was higher during the species feeding activity period (e.g. nocturnal, diurnal). The reason for N excretion being affected by time of day could be because protein metabolization generates ammonia, which is a toxic substance and, therefore, must be excreted rapidly^41^. Compared to N, P metabolization does not generate toxic compounds that must be eliminated shortly after ingestion, and this could be why P excretion was not related to time of day^38^.

Our work was built upon the previous studies of Vanni and McIntyre^14^ and Allgeier et al.^13^ to integrate the MTE and ES frameworks for predicting animals’ nutrient excretion rates, and all three studies found that body size was by far the strongest influence on nutrient excretion. However, these prior studies tested a much wider range of body sizes (1 µg to 500 g dry mass^14^, 0.04 to 2597 g^13^) than body N:P ratio. That disparity could yield a bias in favor of detecting the influence of MTE variables, so we designed our study to focus on a single taxon with a more limited range of body size (0.021 to 22.01 g dry mass) yet similarly variable body stoichiometry. However, we still found that body size is the key control on N and P excretion rates.

Conceptual integration of MTE and ES in this study and others revealed that body size is the key control on nutrient recycling by aquatic animals. Even though our study included a wide range of temperatures, body stoichiometry, and diets, these factors had little detectable influence. While our statistical models provide a useful way to estimate nutrient excretion rates in our study system, they are not a replacement for collecting field data. The mass balance constraints embodied in the ES framework are a fundamental constraint on nutrient recycling, hence researchers seeking accurate estimates of nutrient excretion by aquatic animals should gather direct measurements to verify the applicability of predictive models to their focal species or ecosystem.

## Methods

### Study site and species

The study was conducted at Rio Guapiaçu, (22°26′08.1″ S, 42°45′34.2″ W), a fourth order stream located in the hydrographic complex Guapiaçu-Macacu, inside the Reserva Ecológica de Guapiaçu (REGUA), in Cachoeiras de Macacu, RJ, Brazil. The hydrographic complex supplies water to approximately 2.5 million people in five cities^42^. All fish were sampled from an approximate 100 m long reach containing a mixture of substrates (bedrock, leaf litter and sand patches) and habitat types (run and pool).

We focused on the 12 numerically-dominant fish species in the community, representing 8 families and 4 orders (Table 5). Information on the feeding groups (detritivore, omnivore, invertivore and piscivore) was obtained on published literature^43–46^ and Fishbase (www.fishbase.se), and confirmed by isotope analysis (Fig. S2 in the Supplementary Information).

**Table 5:** List of the studied species with their feeding groups and body size measurements (as estimated by dry weight).

Order	Family	Species	Body size range (g)	Feeding group
Siluriformes	Callichtyidae	Scleromystax barbatus*	0.31–2.05	Omnivore
Siluriformes	Heptapteridae	*Pimelodella lateristriga*	0.18–4.16	Invertivore
Siluriformes	Heptapteridae	*Rhamdia quelen*	0.19–22.01	Piscivore
Siluriformes	Heptapteridae	*Acentronichthys leptos*	0.39–0.45	Invertivore
Siluriformes	Loricariidae	Ancistrus multispinis*	0.93–6.62	Detritivore
Siluriformes	Loricariidae	Rineloricaria sp*	0.16–7.48	Omnivore
Siluriformes	Trichomycteridae	*Trichomycterus sp*	0.26–2.19	Invertivore
Characiformes	Characidae	*Bryconamericus ornaticeps*	0.14–5.30	Invertivore
Characiformes	Characidae	*Mimagoniates microlepis*	0.03–0.49	Invertivore
Characiformes	Crenuchidae	*Characidium vidali*	0.13–0.84	Invertivore
Cyprinodontiformes	Poeciliidae	*Phalloceros harpagos*	0.02 – 0.22	Omnivore
Synbranchiformes	Synbranchidae	*Synbranchus marmoratus*	0.42–10.01	Piscivore

*Armored catfish—fish of the family Loricariidae and Callichthyidae, named for the rows of overlapping bony plates that cover and protect their bodies.

### Nutrient recycling trials

We measured individual excretion rates (µg ind^−1^ h^−1^) of nitrogen and phosphorus. Nitrogen was analyzed as ammonium (NH_4_^+^-N, hereafter N) using fluorometry (Aquafluor, Turner Designs, Sunnyvale, CA, USA) following Holmes et al.^47^ as modified by Taylor et al.^48^. Phosphorus was analyzed as soluble reactive phosphorus (PO_4_^3—^P, hereafter P) using the molybdenum blue method^49^ with an autoanalyzer (Lachat, Zellweger Analytics, Milwaukee, WI, USA).

Fish were collected through backpack electrofishing (LR-24, Smith Root, Vancouver, WA, USA) and placed in a holding chamber in the river for ~ 15 min to acclimate. To begin a trial, one fish was placed in a clean new ziplock bag or an acid-washed translucent plastic box containing a known volume (400–5000 mL, depending on fish size) of fresh stream water that was pre-filtered (200 µm) to remove particles. Plastic bags or boxes were placed at the river margin to maintain temperature and minimize fish stress^38^.

After a 60-min incubation time, we collected a water sample using a 60-mL syringe, and filtered it (GF/F, 0.70 µm pore size, Whatman, Maidstone, Kent, UK) into a new high density polyethylene bottle and placed in a cooler in the field. The samples for NH_4_^+^-N analysis were analyzed within a few hours upon collection in the field, while those for PO_4_^3—^P were frozen until analysis. Supplemental samples from the stream were collected and filtered on each day to correct for background nutrient concentrations.

Each fish was measured (standard length) following the incubation, and most were released unharmed. We estimated its wet mass using a species-specific length-mass regression relationship from previous work (Manna L. unpublished data), and converted to estimated dry mass using a dry:wet ratio of 0.23 derived as an average of observations from this fauna. A subset of 3–5 individuals per species were sacrificed to measure body chemistry (%C, %N, %P).

Excretion trials were conducted during three seasons (summer, fall and winter) between 2016 and 2018, and included both daytime (9am to 4 pm) and nighttime work (8 pm to 12am). Water temperature was measured once daily with a thermometer on the day of most excretion trials. For the days in which temperature measurements were not available, we estimated it using simple linear regression of air and water temperatures (see details in Supplementary Information). Temperatures varied widely (9.9–25.7 °C; Table S1 in Supplementary Information), and our estimation approach for missing temperature data captured the seasonal patterns well.

### Statistical analyses

We tested the effect of body size (log_10_[g dry mass]), temperature (°C), body N:P (molar), (or alternatively armor classification, i.e., presence of well-developed scutes or not), dietary group (as a proxy for dietary nutrient content) and time of day (day or night) on logarithm of excretion rates of N and P. We used linear mixed-effects models (LMEM) with species as a random factor. A question of interest is whether changes in excretion rate in relation to the fixed predictor variables are related to the species and family identity. Consequently, we ran the LMEM with intercept varied among species nested in family factor^50^. We used testing-based procedures with backward elimination to select the best model^50–52^. In these procedures, we started with all the predictors in the model and then removed the predictor with the largest p-value as long as it is greater than 0.05 (critical values). Then, we refit the model and remove the new non-significant predictor with the largest p-value. And so on until the simplest model is determined with all significant predictors. Each pair of models (the most complex vs the simplest) was evaluated with the Chi-square test. The LMEM were generated using the package lme4^50^ and lmerTest^53^ in statistical program R version 4.1.1^54^.

### Ethical approval

All methods were performed in accordance with the relevant guidelines and regulations. All fish in this research were collected following Brazilian laws under the permits numbers 39170 and 64907-1, authorized by Instituto Chico Mendes de Conservação da Biodiversidade (ICMBio/MMA).

## Supplementary Information

Supplementary Information.

## Data Availability

The data that support the findings of this study will be made available as Supplementary Information files that will be freely accessible on nature.com upon publication.
