# Supplementary material for: Body size has primacy over stoichiometric variables in nutrient excretion by a tropical stream fish community
Source: Sci Rep. 2022 Sep 1;12:14844. doi: 10.1038/s41598-022-19149-w (PMC9436996; doi:10.1038/s41598-022-19149-w)
Supplement: Supplementary file 1 — Supplementary Information. [file 41598_2022_19149_MOESM1_ESM.doc]

**Supplementary Information**

Figure S1. Excretion rates of N (a) and P (b) of armored and non-armored fish species in a Brazilian stream.

Figure S2. Result of stable isotopic analysis (d15N) according to fish functional feeding groups.

*
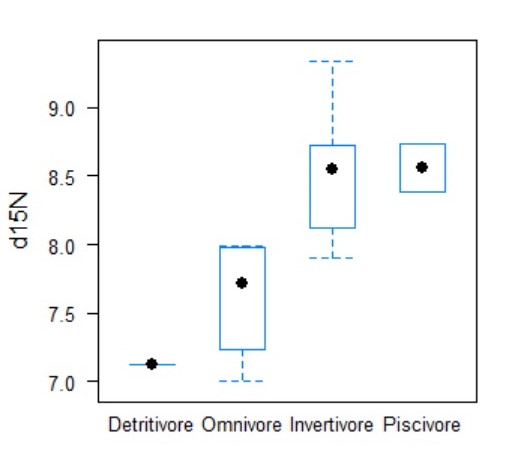
*

*Details on our stream temperature estimates*

The weather station in REGUA, the reserve where our site in Rio Guapiaçu is located, was out of order during our experiments, therefore we do not have air temperature measurements. To obtain an equation to estimate the air temperature in REGUA, we plotted the air temperature from a close weather station (located in Nova Friburgo) in the x axis, with the air temperature data from the weather station in REGUA in the y axis (Figure A2).Air temperature data for the weather station in Nova Friburgo was obtained from INMET (Instituto Nacional de Meteorologia).

We obtained the following equation: y= 1.1589x + 3.8237.

Where:

y= Air temperature in REGUA

x= Air temperature in Friburgo

Figure S3. Relationship between the air temperature at REGUA and in Friburgo.


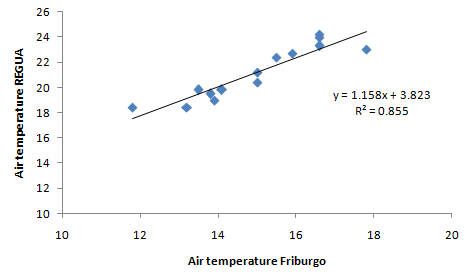


Then, we plotted the estimated air temperature in REGUA in the x axis with the water temperature we measured in the stream in the y axis (Figure A3). This way, we obtained an equation that allows the estimation of water temperature for the days we did not measure it.

We obtained the following equation: y= 1.1719x – 8.0599.

Where:

y= Water temperature in Rio Guapiaçu

x= Air temperature in REGUA

Figure S4. Graph showing the relation between the estimated air temperature in REGUA and the water temperature in Rio Guapiaçu.


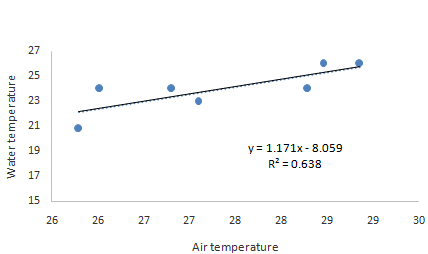


Table S1- Date, season of the year, average, maximum and minimum estimated water temperature during nutrient recycling trials.

| Date | Season | Time of day | Average  temperature (ºC) | Minimum and maximum  temperature (ºC) |
| --- | --- | --- | --- | --- |
| August 2016 | Winter | Day | 22.2 | 22.2 |
| December 2016 | Summer | Day | 25.5 | 25.3 – 25.7 |
| January 2017 | Summer | Day | 23.7 | 22.4 – 25.1 |
| February 2017 | Summer | Day | 23.3 | 23.3 |
| April 2018 | Fall | Night | 24.3 | 24.3 |
| May 2018 | Fall | Night | 15.6 | 9.9 – 21.7 |
